# Palæolithic Osteology

**Published:** 1881-07

**Authors:** J. L. Cilley


### PALÆOLITHIC OSTEOLOGY.

Editors *Lancet* and *Clinic:*

Until of late years the mass of mankind—not excluding the educated classes—held that the earth and man were created simultaneously; moral laws were imposed upon man from the beginning, and that everything was in harmony, until his first disobedience, which occurred in his " beautiful birth-place." We are told that both body and soul, to-day, share the effects of the disruption of that harmony. All this happened about 6,000 years ago. The firmness of these habitual opinions has been tried by the revelations of geology, which are numerous. Our earth has been the seat of vegetable and animal life for an immense period of time. Amongst a multitude of proofs, it is *one* that does the business, as regards the firmness of the above referred to habitual opinions. Palæolithic osteology is that one.

The geologist says man is among the most modern of living creatures; but, although his first appearance is comparatively recent, it is positively remote, anywhere between 20,000 and 100,000 years, taken as a minimum.

Again, if we are the descendents of a common progenitor, man must have existed during a vast period of time. The gulf between the family of man and the family of apes is enormous, but that between the family of man-like apes (the gorilla) and of apes lowest in the scale is even greater.

Man's existence goes back to the period of the post-glacial drift, which indicates an antiquity of tens of thousands of years. Human bones and objects of human manufacture occur in geological relation to the remains of fossil species of elephant, hyena, etc. They certainly belong to a time quite separate from that of history. The drift implements belong to the old stone age. Animals of this period whose bones are found with these rude stone implements, comprise several species of mammalia long since extinct. The scores of centuries of history form but a fraction of the time which has elapsed since the stone implements of prehistoric tribes were first buried under beds of gravel and sand.

These arguments suggest high antiquity—but ages have elapsed since men lived who were advanced enough in the arts to bake brick and pottery. The Valley of the Nile has mud which has been deposited at a rate of probably not averaging more than a few inches in a century. Borings sixty feet deep furnish evidences of the existence of people advanced in the art of pottery. Beyond the historic period of man, there stretches back a vast indefinite series of prehistoric ages.

The palæolithic period is known as the Cave Period, as evidence is furnished of men occupying caves. Skulls and other remains have been found to illustrate the men of this period. Their cerebral development was good, and in intellectual aptitude they averaged well with men of the nineteenth century.

Human bones, together with implements shaped by human workmanship, show that men lived at remote times in France, Belgium,

England, Brazil, Sicily, India and elsewhere. The habits of life of these people were similar to those of the Esquimaux, and the reindeer prevailed, doubtless, in Central France.

Respectfully,

J. L. CILLEY, M. D.

